# NApy: efficient statistics in Python for large-scale heterogeneous data with enhanced support for missing data

**DOI:** 10.1093/gigascience/giaf140

**Published:** 2025-11-06

**Authors:** Fabian Woller, Lis Arend, Christian Fuchsberger, Markus List, David B Blumenthal

**Affiliations:** Biomedical Network Science Lab, Department Artificial Intelligence in Biomedical Engineering, Friedrich-Alexander-Universität Erlangen-Nürnberg, Nürnberger Straße 74, 91052 Erlangen, Germany; Institute for Biomedicine, Eurac Research, A.-Volta-Str. 21, 39100 Bolzano, Italy; Institute for Biomedicine, Eurac Research, A.-Volta-Str. 21, 39100 Bolzano, Italy; Data Science in Systems Biology, TUM School of Life Sciences, Technical University of Munich, Maximus-von-Imhof-Forum 3, 85354 Freising, Germany; Institute for Biomedicine, Eurac Research, A.-Volta-Str. 21, 39100 Bolzano, Italy; Data Science in Systems Biology, TUM School of Life Sciences, Technical University of Munich, Maximus-von-Imhof-Forum 3, 85354 Freising, Germany; Munich Data Science Institute (MDSI), Technical University of Munich, Walther-Von-Dyck Str. 10, 85748 Garching bei München, Germany; Biomedical Network Science Lab, Department Artificial Intelligence in Biomedical Engineering, Friedrich-Alexander-Universität Erlangen-Nürnberg, Nürnberger Straße 74, 91052 Erlangen, Germany

**Keywords:** statistical software, efficient computing and parallelization, Python, large-scale datasets, missing data

## Abstract

**Background:**

Existing Python libraries and tools lack the ability to efficiently compute statistical test results for large datasets in the presence of missing values. This presents an issue as soon as constraints on runtime and memory availability become essential considerations for a particular use case. Relevant research areas where such limitations arise include interactive tools and databases for exploratory analysis of biomedical data.

**Results:**

To address this problem, we present the Python package NApy, which relies on a Numba and C++ backend with OpenMP parallelization to enable scalable statistical testing for mixed-type datasets in the presence of missing values.

**Conclusions:**

Both with respect to runtime and memory consumption, NApy outperforms competitor tools and baseline implementations with naive Python-based parallelization by orders of magnitude, thereby enabling on-the-fly analyses in interactive applications. NApy is publicly available at https://github.com/DyHealthNet/NApy .

## Introduction

Statistical analyses in biomedical research areas often deal with datasets consisting of several thousand samples and variables. Population-based cohort studies, such as the Cooperative Health Research in South Tyrol (CHRIS) study [1] or the Study of Health in Pomerania (SHIP) [2], typically include several thousand study participants. These studies typically encompass diverse health and lifestyle records represented by quantitative (e.g., blood pressure, height), dichotomous (i.e., binary, such as sex, presence of a disease), and categorical variables (e.g., body mass index categories, job). Additionally, due to factors like experimental design or the nonapplicability of certain measurements to specific subgroups of participants, the population cohort data frequently exhibit a significant amount of missing data [3].

While, classically, biomedical data analyses on population cohort data mostly investigate statistical associations between *specific* variables (e.g., correlations between measurements, such as body mass index and blood pressure in population cohorts), the rapidly growing popularity of systems approaches in biomedicine makes it increasingly relevant to be able to efficiently compute pairwise statistical associations for *all* available pairs of variables in a dataset. For instance, one could imagine an (online) data explorer where users can interactively mine population cohort data for networks of variables whose pairwise association strengths differ between two user-selected conditions or contexts. Similar network-based data explorers already exist for precomputed expert-curated networks, for example, CoVex [4] for exploration of the SARS-CoV-2 virus–host–drug interactome or NeDRex [5] and Drugst.One [6] for general-purpose exploration of knowledge graphs defined over biomedical entities such as drugs, diseases, and genes. To leverage the data analysis paradigm exemplified in these approaches for the analysis of context-dependent association networks that must be computed on the fly from population cohort data, a statistics tool is required that combines the following two properties:

- The tool should be runtime- and memory-efficient enough to enable real-time computation of association networks for large-scale datasets (i.e., computation of all pairwise statistical associations between variables).
- The tool should be capable of handling heterogeneous data with high rates of missing values while minimizing information loss (i.e., pairwise data removal instead of complete-case analysis).

As an easily accessible and yet powerful programming language with plenty of available modular extensions, Python has become one of the most popular languages in statistical programming and data mining. Several state-of-the-art Python libraries for statistical analysis exist. While the core Python libraries NumPy [7] and pandas [8, 9] primarily support basic correlation computations, SciPy [10] and Pingouin [11] provide a broader spectrum of statistical functions and distributions. However, all of these libraries exhibit specific limitations with respect to the previously described use case. First, pairwise missing data removal or pairwise feature calculations are restricted to a limited subset of statistical tests. Second, their application to large-scale datasets within an interactive data explorer is impractical due to the prolonged runtime and the lack of inherent parallelization capabilities. This limitation arises primarily because more efficient popular Python statistics libraries, such as SciPy and pandas, are mainly implemented in Cython, which inherently lacks support for intrinsic parallelization.

**Figure 1:**
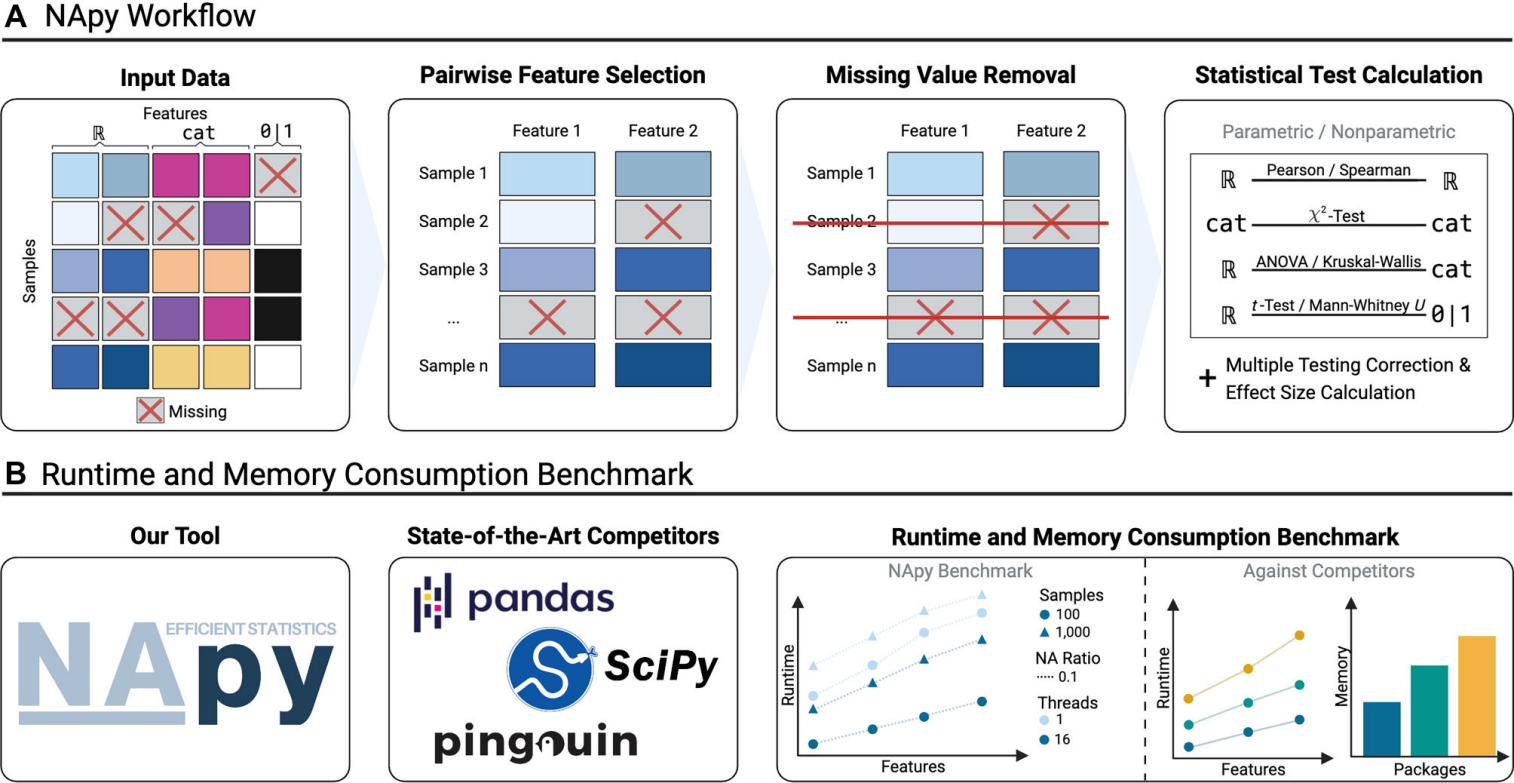
Overview of NApy’s workflow (A) and benchmark analyses of runtime and memory consumption (B). NApy enables efficient computation of standard statistical tests in parallel with pairwise deletion of missing values. Benchmarking on both a simulated and a population-based cohort study demonstrates that NApy outperforms state-of-the-art competitors by orders of magnitude. Created with BioRender.com.

For this reason, we developed our Python tool NApy, which addresses all of the abovementioned issues. We demonstrate the performance of NApy on both simulated data (with varying numbers of features, samples, threads, and missing value ratios) and a real-world population cohort. Our results show that NApy uses memory much more efficiently than most available Python competitors and outperforms state-of-the-art competitors in terms of runtime and memory by orders of magnitude (see Fig. 1).

## Related Work

A range of computational tools in Python, including NumPy [7], SciPy [10], Pingouin [11], and pandas [8, 9], is available for conducting statistical tests, yet each comes with specific limitations concerning our use case (Table 1). NumPy and pandas, two popular Python libraries for data handling and modeling, are largely limited to correlation analyses and thus only support analyses of continuous data. NumPy is restricted to the calculation of Pearson correlation coefficients and lacks pairwise deletion of missing data. Conversely, pandas expands correlation options, providing Pearson, Spearman, and Kendall coefficients across features in the data with pairwise deletion of missing values, but does not provide *P*-values. SciPy and Pingouin, on the other hand, offer a broader selection of statistical tests. Expanding upon NumPy’s core features, SciPy offers additional array computation tools, incorporating efficient, low-level language implementations in Fortran, C, and C++ to enhance processing speed. It provides a range of statistical tests for categorical and continuous data, including two-sided *P*-value reporting. Notably, SciPy enables pairwise feature calculations and missing value deletion specifically for Spearman rank correlation, while SciPy’s other statistical tests lack these capabilities. SciPy further includes multiple testing correction and limited options for effect size calculations. Finally, Pingouin, an open-source statistical package written in Python 3 and based on pandas and NumPy data structures, mostly uses the low-level SciPy functions to provide richer and more exhaustive output. Thus, it includes a wide range of statistical tests with multiple testing correction and effect size calculation directly computed and included in the output pandas DataFrame along with other results, such as the 95% confidence intervals of the difference in means when calculating a *t*-test. It allows computation of pairwise correlations between columns of a pandas DataFrame with specification of a nan_policy parameter for pairwise deletion of missing values. However, as in SciPy, these functionalities are only provided for a subset of statistical tests. A common limitation across all four packages in analyzing large-scale datasets with thousands of features and samples is the lack of built-in parallelization.

**Table 1:** Overview of state-of-the-art Python libraries for statistical tests computation, including NApy.

	NumPy	pandas	SciPy	Pingouin	NApy
Input data	NumPy matrix	pandas DataFrame	NumPy matrix	pandas DataFrame	NumPy matrices, PyTorch Tensors, or pandas DataFrames
Statistical tests	Only $r^2$	Only $r^2$ and ρ	$>$ 20	$>$ 20	7
Pairwise input feature calculation	✓	✓	Only ρ	Only $r^2$ and ρ	✓
Pairwise missing value removal	$\times$	✓	Only ρ	Only $r^2$ and ρ	✓
*P*-value calculation	$\times$	$\times$	✓	✓	✓
Effect size calculation	$\times$	$\times$	Limited	Extensive	✓
Multiple testing correction	$\times$	$\times$	2	5	3
Output format	NumPy matrix ($r^2$)	pandas DataFrame ($r^2$ & ρ)	SciPy object (test statistics and *P*-values)	pandas DataFrame (test statistics, unadjusted *P*-values, effect size, additional results)	dictionary of NumPy matrices, PyTorch Tensors, or pandas DataFrames (test statistics, unadjusted *P*-values, effect sizes)
Parallelization	$\times$	$\times$	$\times$	$\times$	✓

Apart from any considered Python competitors, we also need to mention that, to the best of our knowledge, there do not exist any statistics tools in C++ itself suitable for our use case. The Boost library [12] offers functionality for several statistical tests (e.g., *t*-tests and Pearson correlation) but does not offer the computation of statistical tests for all pairwise feature combinations given a data matrix or missing value removal. Similarly, ALGLIB [13] offers a higher variability of statistical tests (*t*-tests, $\chi ^2$-tests, Mann–Whitney *U* tests, and correlation coefficients) but does not provide functionality for pairwise feature computations and neglects missing value removal—furthermore, it only supports single-threaded computations in its free version.

## Methodology and Functionality

### Overview

NApy consists of seven statistical tests that compute respective statistic values, *P*-values (with support to correct for multiple testing), and effect sizes on given input data matrices in a way that efficiently removes missing values in a pairwise fashion. The implemented tests cover all combinations of continuous, dichotomous, and categorical features, which are the main data types in large biomedical datasets. Additional statistical tests can be integrated by following the guidelines provided in NApy’s GitHub repository and illustrated in Supplementary Fig. S1.

All tests are implemented in C++, parallelized with OpenMP, and integrated into a Python wrapper by using pybind11 [14]. Additionally, for each test, we offer an analogous implementation based on the Python just-in-time compiler Numba [15]. As shown below, the C++ backend is more memory-efficient and faster for nonparametric tests. The Numba backend is faster for parametric tests and easier to extend for potential future contributors without knowledge of C++. An essential advantage of both implementations is that we perform pairwise missing value removal on the fly during statistics computations instead of outsourcing this subsetting procedure as a “preprocessing” step.

### Pairwise missing data removal

One common approach to deal with missing data is to simply restrict to those samples in the data that are fully present (complete-case analysis). However, especially for large datasets and in the presence of high percentages of missingness, this leads to the loss of a significant amount of potentially valuable information [16]. On the other hand, standard imputation techniques, such as *k*-nearest-neighbor classification, regression imputation, or random forest models, provide possibilities to infer missing values [17]. However, the choice of an optimal imputation method can be highly dataset-dependent, and downstream analyses can be quite sensitive to the chosen imputation [18]. An alternative that does not suffer from potential distortion of imputation, but also does not waste as much information as the complete-case analysis, is pairwise missing data removal, as implemented in several statistical software libraries (e.g., with the option nan_policy=’omit’ in the SciPy library function scipy.stats.spearmanr [10]).

We briefly want to formalize the concept of pairwise missing data removal as it is implemented in NApy: assume we are given some input data matrix $\mathbf {X} \in \mathbb {R}^{F, S}$ consisting of *F* features and *S* samples. Features can be of any type: quantitative, dichotomous, or categorical, with categories assumed to be label-encoded (i.e., with labels as integers starting from zero). Furthermore, we assume that missing data entries are encoded by some special value, which we will abbreviate by *m*. For a given pair of features $\left(g, h\right)$ with $g, h \in \mathbb {R}^{S}$ (i.e., two rows from $\mathbf {X}$), we want to perform a statistical association analysis. Pairwise missing data removal now means that, for a given feature pair $\left(g, h\right)$, we extract the subset of paired sample entries $\mathcal {I}(g,h)$, where at both positions in *g* and *h*, the corresponding value is non-missing. More formally, we extract the set of pairs $\mathcal {I}(g, h) := \left\lbrace \left(g_i, h_i\right) \bigl \vert \ g_i \ne m \wedge h_i \ne m;\ i=1,\dots ,S \right\rbrace$, which is then given as input to the respective statistical test. Note that the actual size of the input set $\mathcal {I}$ depends on the chosen pair of features and on the location of the respective missing data entries. In particular, it may happen that $\mathcal {I}$ is empty.

### Input

NApy’s statistical tests are designed to operate on 2-dimensional data structures, including numpy.ndarray objects [7], PyTorch tensors [19], or pandas.DataFrame objects [8, 9]. Depending on the respective datatype combination, the test functions expect either one matrix storing only continuous, dichotomous, or categorical feature values or two input matrices when handling a mix of continuous and categorical or dichotomous feature variables. Independent of the input format, the users can specify a special floating point value indicating missing data as a parameter. Furthermore, the users can specify which input dimension of the matrices should be interpreted as samples and which as features. The number of threads to use in parallel computations of results can also be set. Additionally, users can choose between the underlying C++ or Numba implementations. Finally, the users can specify a list of desired data matrices to be returned, such as unadjusted and corrected *P*-values, respective statistics values, and a selection of effect sizes. A representative usage example of the Pearson correlation function is shown in Listing 1.

### Output

For each supported test, the output is represented by a Python dictionary that stores data matrices with the user-requested output (*P*-values, adjusted *P*-values, and/or effect sizes). We store output matrices as 2-dimensional objects of the same type as the input data (i.e., as NumPy arrays, PyTorch tensors, or pandas DataFrame objects). NApy’s output is directly interoperable with popular machine learning frameworks such as scikit-learn or PyTorch, as exemplified in the examples in NApy’s GitHub repository and in Supplementary Listings 1 and 2.

In the case of continuous-continuous and categorical-categorical tests, any returned output matrix $\mathbf {M} \in \mathbb {R}^{F, F}$ for *F* features in the input data matrix is quadratic and stores at position $\mathbf {M}_{i,j}$ the associated result value for features $i, j \in \left\lbrace 1,\dots ,F\right\rbrace$. In the heterogeneous case of categorical-continuous input data for $F_{\text{C}}$ categorical and $F_{\text{Q}}$ quantitative input features, such a returned output matrix $\mathbf {M} \in \mathbb {R}^{F_{\text{C}}, F_{\text{Q}}}$ stores at position $\mathbf {M}_{i,j}$ the corresponding result value for categorical feature $i \in \left\lbrace 1, \dots , F_{\text{C}}\right\rbrace$ and quantitative feature $j \in \left\lbrace 1, \dots , F_{\text{Q}}\right\rbrace$. The dichotomous-continuous input and output are defined analogously.

Within our tool, output matrices can store unadjusted *P*-values, adjusted *P*-values (using Bonferroni [20], Benjamini–Hochberg [21], or Benjamini–Yekutieli multiple testing correction [22]), values of the respective test statistic, or suitable effect sizes (Table 2). Note that when applying multiple testing correction for the homogeneous input-type case, we do not count “self-tests” of features on the diagonal as actually performed tests and therefore set $\mathbf {M}_{i,i} \leftarrow \mathtt {numpy.nan}$ for all features $i \in \left\lbrace 1, \dots , F\right\rbrace$ in any of the multiple testing corrected output matrices $\mathbf {M}$.

**Listing 1.**
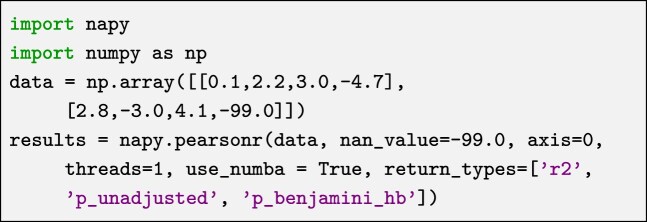
Example usage of NApy’s Pearson correlation library function.

**Table 2:** Overview of NApy’s output values for each test.

Test	Statistic	Effect size(s)	*P*-value(s)
Pearson		$r^2$	Unadjusted and
Spearman		*P*	corrected
$\chi ^2$ -Test	$\chi ^2$	$\phi$ , Cramer’s *V*	(Bonferroni, Benjamini–Hochberg, Benjamini–Yekutieli)
*t*-Test	*t*	Cohen’s *D*	
Mann–Whitney *U*	*U*	Pearson *r*	
ANOVA	*F*	Partial $\eta ^2$	
Kruskal–Wallis	*H*	$\eta ^2$	

### Implementational details

#### Parallelization and missing data removal

All implemented tests have in common that parallelization is employed at the outer level of pairwise feature analysis. That is, the set of all pairs of (continuous, dichotomous, or categorical) features given some input data matrix (or matrices) is distributed among the number of user-specified threads, which are generated by OpenMP in the C++ implementation or Numba directly. Hence, in both cases, parallelization runs thread-based on shared memory. No memory needs to be multiplied or copied among processes. For a given pair of features, pairwise missing value removal is run in combination with the necessary statistical computations. That is, only paired sample entries with present data entries at both positions are kept as input for further calculations. In the following, we briefly sketch individual details for each supplied statistical test.

#### Pearson correlation

Two-sided *P*-values for a given pair of features are computed based on the cumulative distribution function (CDF) of the $\beta$-distribution. If the number of remaining sample pairs (i.e., the cardinality of set $\mathcal {I}$) is less than 2 after pairwise missing data removal, the corresponding *P*-value is not defined and reported as numpy.nan. If the number is at most 1, both $r^2$ and the *P*-value are not defined and returned as numpy.nan.

#### Spearman correlation

First, data are sorted and ranked in parallel for all features to avoid costly sorting operations in the later pairwise processing of features. This allows only accounting for missing data removal in the pairwise feature processing, which can be implemented in linear time. We account for ties in the data by averaging over tied ranks. Two-sided *P*-values are computed based on the CDF of the *t*-distribution. Similar to Pearson correlation, a remaining sample count of at most 2 renders *P*-values undefined, and a count of at most 1 results in both the *P*-value and $\rho$ being undefined.

#### $\chi ^2$ -test

*P*-values are calculated based on the survival function of the $\chi ^2$-distribution. If any of the previously existing categories become empty after the removal of pairwise missing data, the resulting $\chi ^2$-value (along with all other potential return values) is undefined and therefore returned as numpy.nan. In case only one category group exists for one of the two categorical features, *P*-value and Cramer’s *V* are undefined and returned as numpy.nan.

#### *t*-test

For 2-sample *t*-tests, we offer the functionality to let the user choose between applying Student’s *t*-test or Welch’s *t*-test. The difference is that the latter does not assume the variances of the two populations induced by the dichotomous feature to be equal. Either way, two-sided *P*-values are computed based on evaluating the survival function of the *t*-distribution. If, after pairwise missing value removal, the number of remaining samples in one of the two populations is less than 2, or the sum of remaining samples in both categories is less than 3, the *t*-statistic is undefined and hence returned as numpy.nan (as well as all other return types). For Student’s *t*-test, the same holds true in case the pooled standard deviation should equate to zero. In Welch’s *t*-test, if the sum of the two population variances is zero, Cohen’s *D* is not well defined and returned as numpy.nan.

#### Mann–Whitney *U* test

To minimize costly sorting operations during each pairwise analysis of features, each thread initially sorts one continuous variable and subsequently computes test statistics for all dichotomous variables, while accounting for their respective missing values. This way, each continuous variable is sorted only once. We account for ties in the remaining data by averaging over tied ranks and additionally employ the commonly used tie correction for the computation of *z*-values. Following the SciPy implementation scipy.stats.mannwhitneyu, we offer the possibility to compute *P*-values in an exact or asymptotic way. In the asymptotic mode, *P*-values are computed based on evaluating the survival function of the standard normal distribution. In the exact mode, *P*-values are computed by using a dynamic programming approach presented in [23], which is only feasible for smaller input populations. Therefore, we follow the SciPy implementation and offer an auto mode, which automatically chooses between asymptotic and exact mode: if there are no ties for a specific pair of features after missing value removal and any of the two populations has fewer than 8 associated samples, the exact mode is chosen for *P*-value calculation. Otherwise, the asymptotic mode is chosen. If one of the two categories is empty after pairwise missing value removal, the test statistic is not well defined, and we return numpy.nan.

#### Analysis of variance

Using analysis of variance (ANOVA) on mixed-type categorical-continuous data, two-sided *P*-values are computed based on the survival function of Fisher’s *F*-distribution. As with *t*-tests, if one of the categories after pairwise missing data removal is empty, the test statistic is not well defined, and numpy.nan is returned for all return types. Furthermore, if variances within categories are all zero, we additionally check if all group sums are equal and, if not, return an *F*-statistic value of numpy.infty and a zero *P*-value. Otherwise, *F*-statistic and *P*-value are undefined and returned as numpy.nan.

#### Kruskal–Wallis test

Similar to the Mann–Whitney *U* test, continuous variables are presorted prior to performing pairwise statistical tests with all categorical variables. Ties are treated by averaging over tied ranks, and tie correction is used for the computation of the *H*-statistic. In the pairwise feature analysis, after pairwise removal of missing data, we compute two-sided *P*-values based on evaluating the survival function of the $\chi ^2$-distribution. If one of the induced categories is empty after pairwise missing value removal, the test is invalid, and numpy.nan is returned for all possible return types. If the number of remaining samples is less than or equal to the number of present categories in the categorical feature, the partial $\eta ^2$-value is undefined and returned as numpy.nan.

### Verification of correctness

We implemented a series of Python-based unit tests to validate the correctness of computed test statistics, *P*-values, and effect sizes. Each test statistic and corresponding *P*-value calculation was benchmarked against results from the SciPy library and equivalent R libraries, such as Hmisc [24] and the R stats [25] package, using the Python-R-bridge-package rpy2 [26]. For all statistical tests, we tested universally applicable edge cases such as the correctness of the missing value removal functionality, parallel execution, and axis parameter use. Additional tests focused on data type–specific edge cases, such as single-category data or data without any categories after missing value removal. Finally, effect size calculations and multiple testing corrections were similarly verified against equivalent R implementations with effectsize [27], rstatix [28], and lsr [29]. For detailed information on the specific unit tests, please refer to our GitHub repository.

### Supported system architectures

NApy has successfully been installed and run on Linux PCs and servers (Ubuntu 22.04.5, conda 24.9.1) and Mac PCs (macOS Sonoma, conda 24.9.2). For detailed installation instructions on both Linux and Mac, we refer to our GitHub repository.

## Benchmarking

### Setup

#### Overall approach and computing environments

Given our focus on large, mixed-type datasets with a high prevalence of missing values, we assessed runtime and memory usage of NApy, alongside leading state-of-the-art competitors (Table 1). We used simulated data matrices and the CHRIS study data [1]. All benchmarks were performed on a server within Eurac’s compute cluster, equipped with two Intel Xeon Silver 4216 CPUs, each operating at 2.10 GHz with 16 physical cores, enabling a total of 64 threads. To assess possible hardware-specific effects, comparisons of NApy’s Numba and C++ backends were additionally performed on a server hosted at the Erlangen National High-Performance Computing Center (NHR@FAU) with two Intel Xeon Gold 6326 (“Ice Lake”) CPUs, each operating at 2.9 GHz with 16 physical cores (allowing for a total of 32 threads).

#### Simulated data

For the first part of our benchmarks, input data matrices of varying sizes were simulated at random. A simulated data matrix $\mathbf {X} \in [0,1]^{F, S}$ with quantitative values is generated by uniformly sampling values from the unit interval [0,1], with $S \in \lbrace 250, 500, 750, 1000\rbrace$ being the number of samples and $F \in \lbrace 250, 500, 750, 1000\rbrace$ being the number of features. In the case of categorical data, entries in the matrix represent label-encoded categories, with indices taken from $\lbrace 0, ..., c-1\rbrace$, with *c* being the number of categories for a given feature. We ensured that each feature row contains each possible category at least once. In our benchmark data, we fixed the number of categories for each row to $c = 4$.

Given the frequent occurrence of missing values in biomedical data, a predetermined proportion of missing values was introduced at random positions within each feature across both data types, thereby simulating a missing completely at random (MCAR) mechanism. Missing values were represented using a designated floating-point value. As real-world biomedical datasets are typically affected not only by MCAR but also by missing at random (MAR) and by missing not at random (MNAR) mechanisms, we further generated MAR and MNAR patterns using the default settings provided by the MissMecha package [30]. Unless stated otherwise, the presented results refer to MCAR simulations, except for analyses where we explicitly compared the effects of the different missingness mechanisms.

For statistical tests that require a single data type, such as Pearson, Spearman, or $\chi ^2$-test, a single data matrix was simulated. For tests requiring both quantitative and categorical or dichotomous data, separate data matrices with matching sample and feature dimensions were simulated.

#### Real-world data

**Figure 2:**
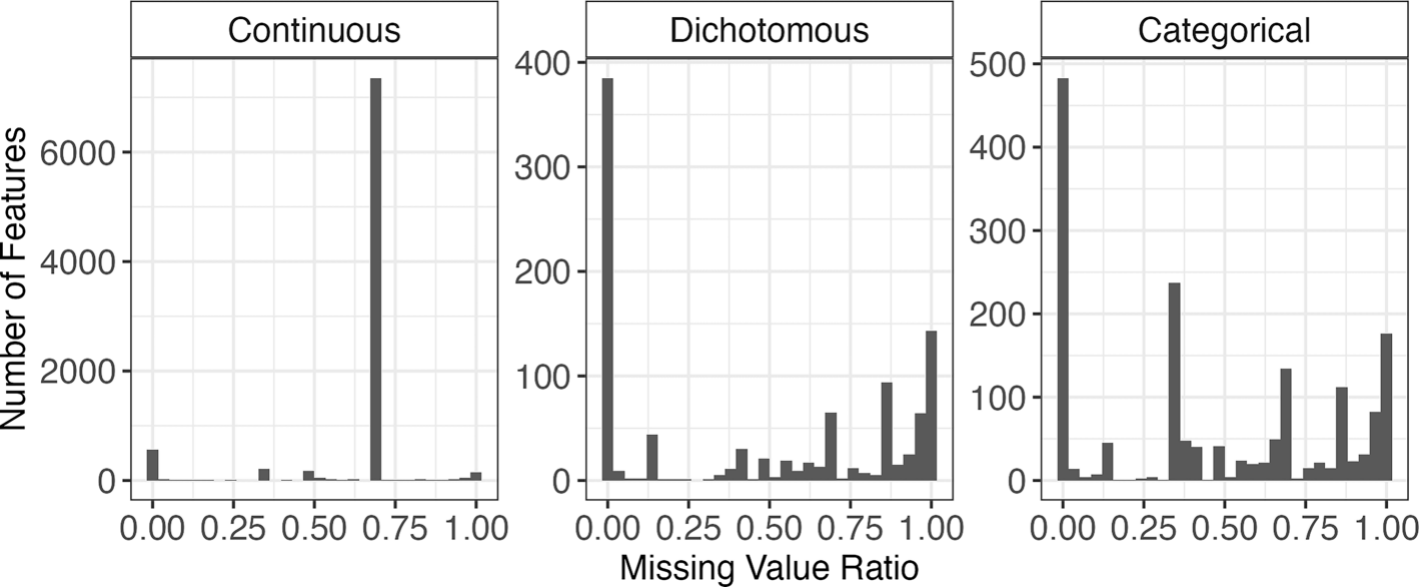
Distribution of missing values in the CHRIS study for continuous, dichotomous, and categorical features.

As a real-world data scenario, we used the CHRIS study data, a population-based study cohort of 13,393 adults recruited from 13 municipalities in the Alpine Val Venosta/Vinschgau district in the Bolzano–South Tyrol province of northern Italy. Baseline visits were conducted from 2011 to 2018, collecting sociodemographic, health, lifestyle, and exposure data from questionnaires, interviews, and instrumental examinations, as well as urine and blood samples for biobanking, DNA extraction, and molecular characterization (genome and exome sequencing, metabolomics, and proteomics). We used the CHRIS study data, consisting of 10,464 features in total. Specifically, 8,803 features are continuous, and 1,661 are categorical, among which 1,010 features are dichotomous. Additionally, as shown in Fig. 2, a substantial amount of missing data is present in the dataset, primarily resulting from instances where samples were not collected from all patients or questionnaires were not answered by all patients.

#### Competitor tools

Not all state-of-the-art libraries offer the functionalities specific to our application, such as statistical test calculation on all feature pairs and pairwise missing data removal and parallelization options. Accordingly, different libraries were selected as competitors, depending on the statistical test, and manual adaptation was necessary to accommodate the specific use case.

The only statistical tests that could be applied directly without modification were Spearman correlation in SciPy (scipy.stats.spearmanr) and Spearman and Pearson correlation in both Pingouin (pingouin.rcorr) and pandas (pandas.DataFrame.corr). However, unlike NApy, none of these libraries inherently support parallelization. Consequently, two variants of a Python-based baseline were included for both Pearson and Spearman correlation, using the correlation coefficient implementations of SciPy and pandas.

No existing tools could process complete data matrices for all other tests to compute pairwise test statistics and *P*-values. Consequently, for the $\chi ^2$-test, the *t*-test, the Mann–Whitney *U*, ANOVA, and the Kruskal–Wallis tests, NApy was compared only against a Python-based baseline implementation using the corresponding functions from the SciPy stats module (chi2_contingency, ttest_ind, mannwhitneyu, f_oneway, kruskal).

Our “naive” Python-based baseline implementation consists of generating all feature pairs with the help of the Python library itertools and performing iterative calculations using NumPy data structures. We employed joblib for parallelizing, enabling direct comparison with NApy’s runtime performance at varying thread counts. In all cases, missing values had to be removed for each feature pair prior to testing. Additionally, certain statistical tests required specific preprocessing: the calculation of contingency tables for the $\chi ^2$-test and the construction of lists of continuous values per category for the *t*-test, the Mann–Whitney *U*, ANOVA, and the Kruskal–Wallis tests. Overall, the manual effort required to adapt competitor tools to our use case was largely comparable across libraries and test types.

It is important to note that the output of the compared competitors and NApy exhibits certain differences. For NApy, we computed and returned statistical values and unadjusted *P*-values, as we intended to stay consistent with the output of the competitor SciPy and Pingouin, which we integrated into the benchmarks of all our tests. In contrast, the pandas.DataFrame.corr function only returns a matrix containing correlation values.

#### Measuring runtime and memory consumption

The runtime for each statistical test and library was measured using Python’s time module, while the allocated memory of the different implementations was measured using Memray [31]. Since Memray can only measure allocated memory within a single process, Python-based competitors employing process-level parallelization were excluded from this benchmark. Because of this, we additionally conducted peak resident memory benchmarks using the Python profiling tool psutil [32], which allows monitoring of memory usage also across multiple processes. Importantly, as stated before, runtime and memory allocation of the competitor pandas were exclusively measured for computing and returning correlations, as pandas does not return *P*-values.

### Results on simulated data

#### Runtime and memory of NApy’s C++ and Numba implementations

**Figure 3:**
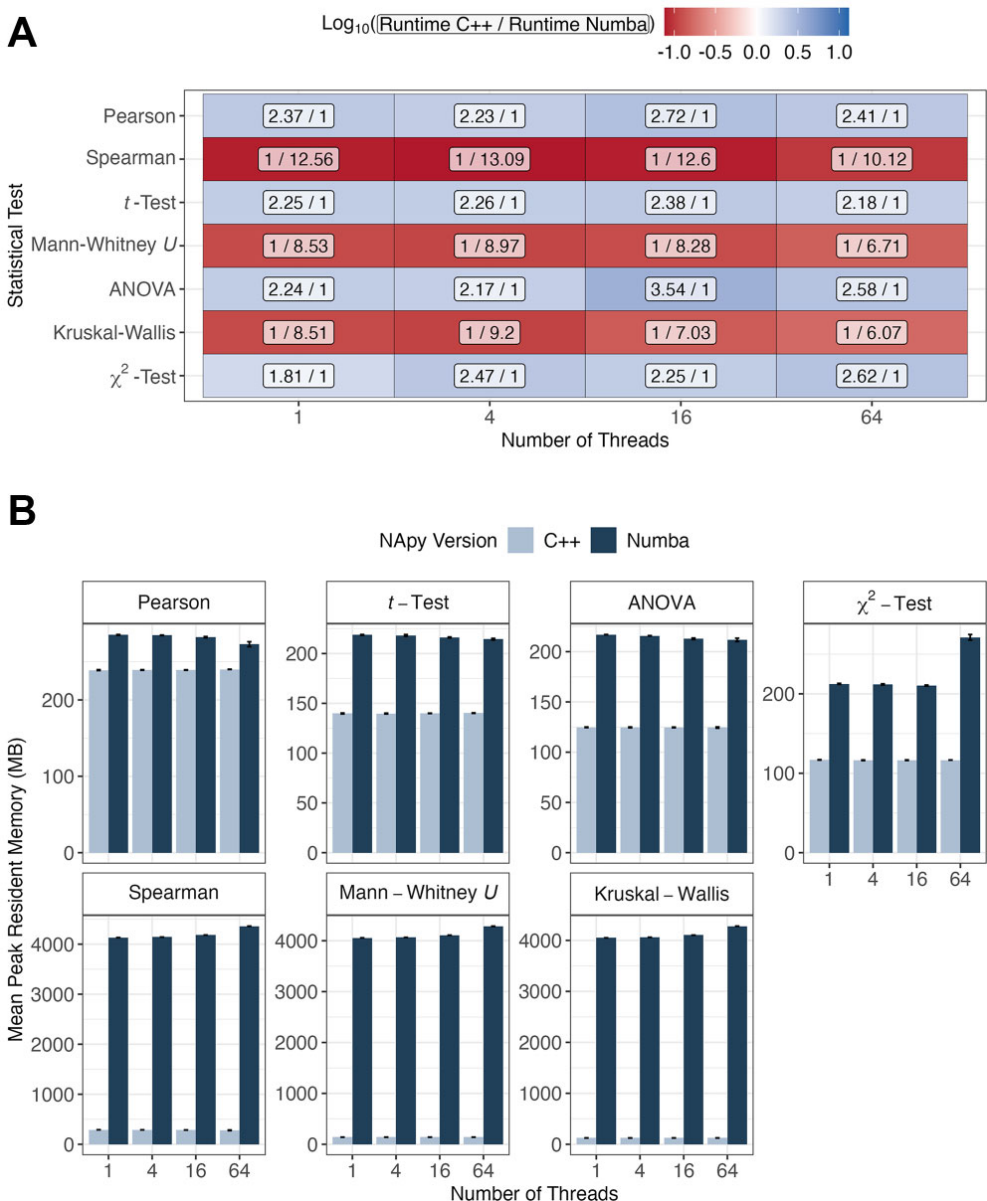
Runtime and peak resident memory evaluation of NApy’s C++ and Numba implementation on Eurac’s compute cluster. Statistical tests were performed using varying numbers of threads on datasets comprising 1,000 samples and features with 10% of missing values per feature. (A) Fold changes between both NApy implementations were computed on the average runtime of 3 independent runs per statistical test and thread count. Cell colors represent the $\log _{10}$-transformed fold changes between NApy’s C++ and Numba implementations, while the cell labels display the corresponding nonlogarithmic fold change values as fractions. (B) For each statistical test and implementation, peak resident memory consumption (in MB) was averaged over 3 runs, with standard deviations represented as error bars.

For all implemented statistical tests, NApy provides the flexibility to execute computations using either the C++ implementation with OpenMP parallelization or the Numba-parallelized version. This functionality is controlled via the parameter use_numba, which is integrated into all statistical test functions. We evaluated the runtime of both implementations and compared them by calculating fold changes between the C++ and Numba-based implementations for all statistical tests. The Numba-based implementation was observed to be faster for parametric tests, while C++ code was faster for nonparametric tests (Fig. 3A).

From a computational perspective, all implemented parametric tests mainly involve a linear scan over the input data structures, while nonparametric tests rely on rank-based computations, which involve computationally more expensive sorting operations and larger data structures. In our peak resident memory benchmarks, we observed that, for all nonparametric tests, the Numba implementation has a substantially higher memory footprint than the C++ version (Fig. 3B). This memory gap between Numba and C++ implementations was much less pronounced for parametric tests. Together with the results of the runtime comparison and in view of the fact that both the runtime and the memory results can be reproduced under different computing environments (Supplementary Fig. S2), this suggests that the C++ implementation is capable of handling complex sorting operations and corresponding data structures more efficiently than Numba.

As default, NApy uses the implementation we observed to be faster for each test (i.e., the C++ implementation for nonparametric tests and the Numba implementation for parametric tests). From this point onward, references to NApy always correspond to the respective default and hence faster implementation.

#### Runtime of NApy versus competitors

**Figure 4:**
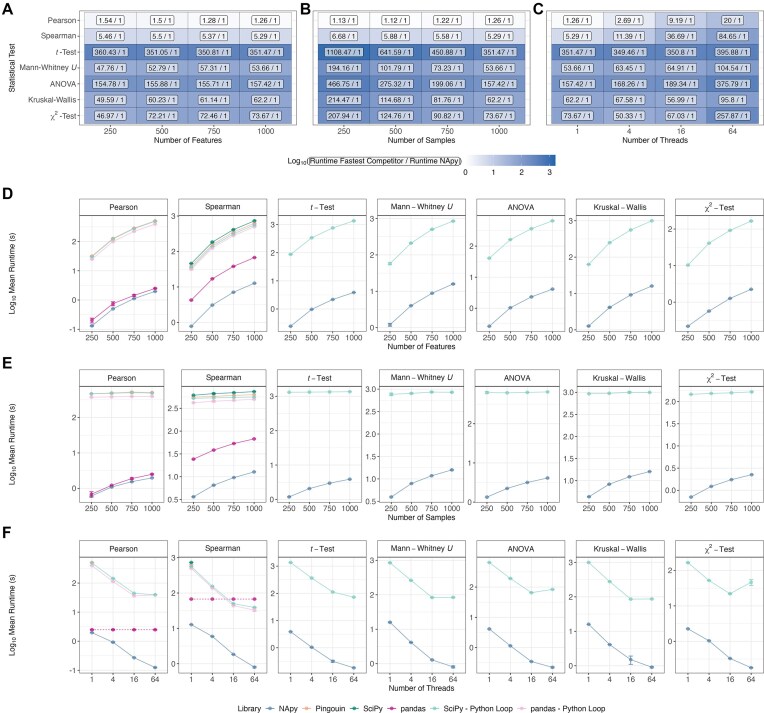
Runtime benchmarks of NApy and applicable competitors across features, sample, and thread counts. (A–C) Fold changes between the runtime of the fastest competitor (pandas for correlations and SciPy-Python Loop for the remaining tests) and NApy were calculated across the different configurations of features (A), samples (B), and threads (C). Cell colors represent the $\log _{10}$-transformed fold changes, while the cell labels display the corresponding nonlogarithmic fold change values as fractions. (D) The runtime (in seconds) was measured on a single thread for datasets with 1,000 samples and varying number of features. (E) Datasets with 1,000 features were used to investigate the sample effect on runtime, with computations being performed on a single thread. (F) Datasets of 1,000 samples and features were simulated, and statistical tests were performed on a varying number of threads. As pandas does not support intrinsic parallelization, the dashed line represents its runtime on a single thread. All datasets included 10% of missing values per feature, and the runtimes were averaged over 3 independent simulation runs. Standard deviations were calculated on the original scale, and both the mean and the mean $\pm$ standard deviation were then $\log _{10}$-transformed for display as points with error bars. The nonlogarithmic NApy runtimes are reported in Supplementary Table S1.

We evaluated NApy’s runtimes against available competitors using simulated datasets with varying numbers of features and samples, performing computations across different thread counts (Fig. 4). For all tests and all numbers of features, samples, and threads, NApy is consistently faster than all tested competitors (Fig. 4A–C). In most of the tested configurations, the speedup is massive; in the most extreme case, NApy is more than 1,000 times faster than its fastest competitor (Fig. 4B).

The only scenarios where comparable runtimes can be achieved with competitor tools are correlation calculations on a single thread (first two rows in Fig. 4A, B). Here, the fastest available competitor is pandas, which, especially for computing Pearson correlation coefficients, is only slightly slower than NApy. Moreover, when NApy is allowed to run in several threads, it clearly outperforms all competitors also for the correlation coefficient computations (first two rows in Fig. 4C), highlighting its improved scaling behavior in comparison to existing tools.

Unsurprisingly, we observed that the number of features has a greater impact on runtime compared to the number of samples (Fig. 4D, E), aligning with expectations since an increase in features leads to a quadratic increase in the number of pairwise statistical tests. Similarly, thread count significantly impacts runtime, with higher thread counts reducing runtime as anticipated due to well-scaling parallelization (Fig. 4F).

#### Runtime under varying missing value ratios

**Figure 5:**
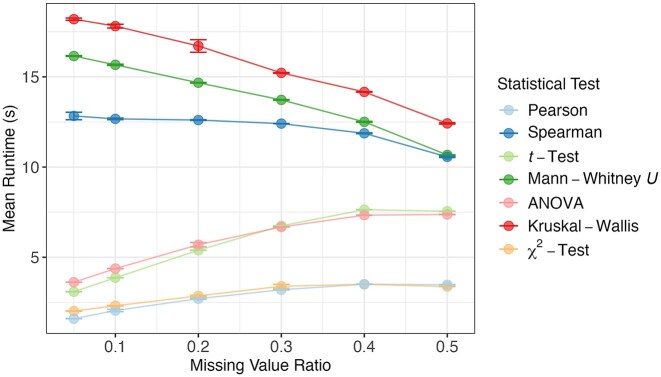
Benchmark analysis of the impact of the ratio of missing values per feature on the runtime of statistical tests in NApy, with missingness simulated under the MCAR mechanism. Simulated datasets with fixed sizes of 1,000 features and 1,000 samples were analyzed to evaluate the effect of varying levels of missing values per feature on runtime. All computations were performed using a single thread, and runtime measurements were averaged over 3 runs, with standard deviations represented as error bars.

Another relevant dataset characteristic influencing the number of available data entries is the proportion of missing data. We assessed its impact by measuring runtime of NApy across varying rates of missing values per feature, using fixed-sized datasets with 1,000 samples and features. The analysis revealed that, for parametric tests, runtime increased with the proportion of missing values in the dataset, whereas for nonparametric tests, an inverse relationship was observed (Fig. 5). This behavior can mainly be attributed to the fact that the sorting and ranking routines of the nonparametric tests rely on more elaborate data structures, which become smaller and cheaper to maintain as the number of available data points decreases. Results are similar for other missingness mechanisms (Supplementary Fig. S3), indicating that NApy’s runtime dynamics are largely independent of the missingness mechanism.

#### Memory of NApy versus competitors

**Figure 6:**
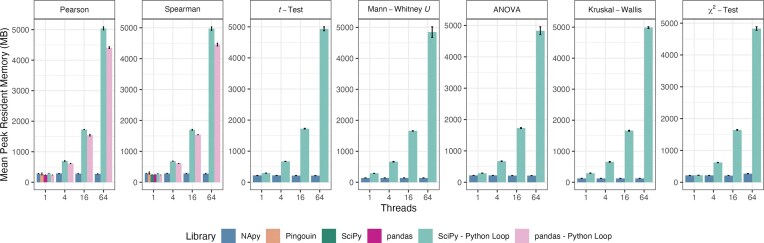
Peak resident memory usage of NApy and its competitors across all statistical tests, evaluated with varying numbers of threads on datasets containing 1,000 samples and features, each with 10% of missing values per feature. For each statistical test and implementation, peak resident memory consumption (in MB) was averaged over 3 runs, with standard deviations represented as error bars.

We benchmarked the allocated memory usage of NApy in comparison to competitors for a fixed dataset size of 1,000 features and 1,000 samples with a fixed ratio of missing values per feature of 10% and varying number of threads used by NApy (Supplementary Table S2). Our measurements show that, except for the computation of Pearson correlation coefficients (which is anyway rather cheap in terms of absolute memory consumption), NApy is clearly more memory-efficient with respect to allocated memory than all other tested tools. Due to the shared-memory parallelization based on OpenMP and Numba, the allocated memory consumption of NApy remains unchanged even when more than one thread is used. Additionally, we analyzed peak resident memory usage of NApy in comparison to Python competitors on simulated datasets of the same sizes as for allocated memory (Fig. 6). Our results show that NApy is mostly on par with available Python competitors in the nonparallelized case (except for slightly increased memory on Numba-based implementations). With increasing numbers of threads (or processes in the case of Python competitors), the memory usage of NApy again remains stable while competitors suffer from a significant memory increase due to process-based parallelism.

### Results on real-world data

**Figure 7:**
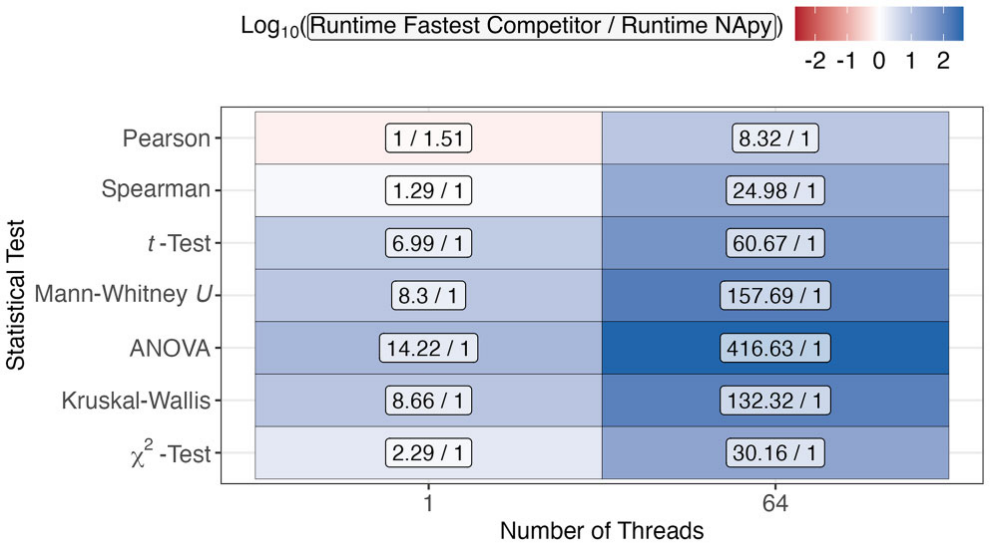
Runtime evaluation of NApy and its fastest competitor on the CHRIS study data. The comparison includes pandas for correlation tests and SciPy–Python Loop for other statistical tests. Fold changes were derived from the average runtimes of 3 independent runs for each statistical test and thread count. Cell colors represent the $\log _{10}$-transformed fold changes, while the cell labels display the corresponding nonlogarithmic fold change values as fractions.

To demonstrate the practical applicability of NApy on a real-world dataset, all statistical tests were performed on the CHRIS study data both without any parallelization (single thread) and with heavy parallelization (64 threads). For correlations, pandas was used as comparison as it was the fastest compared to other competitors on the simulated data (Fig. 4). The results show that, for computing the Pearson correlation using a single thread, pandas slightly outperformed NApy in terms of runtime efficiency (Fig. 7). However, due to pandas’ lack of parallelization capability, NApy achieved significantly improved runtime performance when utilizing 64 threads, with fold changes of 8.32 and 24.98 observed for Pearson and Spearman correlations, respectively. For the remaining statistical tests, the runtime of SciPy–Python Loop was compared to that of NApy, and we observed that, on a single thread as well as on 64 threads, NApy consistently achieved better runtimes than SciPy. Specifically, with NApy, runtime improvements ranged from approximately 2 to 14 when using single threads and even reached maximum values of over 400 using 64 threads. This emphasizes the massive speedup that NApy allows on large datasets such as the CHRIS study data.

## Limitations

The first and probably most obvious limitation of NApy is the number of statistical tests that are currently implemented, especially in contrast to well-established Python statistic libraries such as SciPy and Pingouin. NApy is currently focused on the most commonly employed statistical tests, such that it can already be used in practice for the analysis of mixed-type data in biomedical data analysis and beyond. The application of statistical tests accounting for covariates would be beneficial for analyses involving population-based cohort data. We provide a step-by-step workflow to facilitate the independent integration of new tests into NApy by external researchers, as outlined in the README on GitHub and in Supplementary Fig. S1.

Moreover, one has to note that in direct comparison to, for example, the Pingouin library, NApy currently does not offer the possibility to return as much (meta-)information on statistical results. Yet, we believe that for users working on such large-scale datasets with the focus on efficiency and speed, our current output in the form of statistics values, effect sizes, and (multiple-testing corrected) *P*-values offers a rich source of information well-suited for data analysis focused on especially large datasets.

Lastly, while NApy provides efficient shared-memory parallelization for statistical testing on medium- to large-scale datasets, its scalability is inherently limited by the memory and CPU resources of a single machine. For datasets that surpass these constraints, for example, by including millions of variables, distributed computing frameworks such as Apache Spark [33] or Dask [34] may offer a more suitable solution by enabling scaling across multiple machines. Future work could therefore focus on extending interoperability with distributed systems.

## Conclusions

In this article, we have presented our Python tool NApy, which is capable of performing statistical tests on large heterogeneous input data in the presence of missing data efficiently in parallel. Existing Python-based statistics tools lack the functionality to compute statistical tests on large input data while accounting for pairwise missing data removal in parallel. This becomes an issue as soon as data analyses on heterogeneous large-scale datasets need to be run in a time-critical context.

On both simulated and real-world population-based cohort data, we show that NApy consistently outperforms Python competitors and competing Python baseline implementations in terms of runtime efficiency and memory allocation. An exception arose in the computation of Pearson correlation using a single thread, where pandas showed reduced memory allocation on simulated data and faster execution on cohort data. It is important to note, however, that pandas only computes correlation coefficients, whereas NApy also performs *P*-value calculations, which were not factored into the runtime and memory allocation comparisons.

We are convinced that NApy will enable the development of new real-time statistical analyses on large datasets stemming from experimental measurements that are currently still limited to analyses on precomputed statistical evaluations.

## Availability of Source Code and Requirements

- Project name: NApy
- Project homepage: https://github.com/DyHealthNet/NApy
- License: GPL-3.0 license
- SciCrunch RRID: SCR_027453
- bio.tools ID: https://bio.tools/napy
- System requirements
- Operating system: tested on Ubuntu, macOS
- Programming language: Python and C++
- Package management: Conda
- Hardware requirements: verified to run on a laptop with 12 CPUs and 16 GB RAM
- Benchmarks:
https://github.com/DyHealthNet/NApy_benchmark
- License: GPL-3.0 license

## Supplementary Material

giaf140_Supplemental_Files

giaf140_Authors_Response_To_Reviewer_Comments_Original_Submission

giaf140_GIGA-D-25-00244_Original_Submission

giaf140_GIGA-D-25-00244_Revision_1

giaf140_Reviewer_1_Report_Original_Submission Feng Li -- 7/25/2025

giaf140_Reviewer_1_Report_Revision_1 Feng Li -- 10/24/2025

giaf140_Reviewer_2_Report_Original_Submission Zane Fink, PhD -- 8/8/2025

giaf140_Reviewer_2_Report_Revision_1 Zane Fink, PhD -- 10/27/2025

## Data Availability

To reproduce results for the CHRIS data, access to CHRIS study data is required, which, due to privacy protection rules, has to be formally requested for clearly defined research via the CHRIS Portal [35].

## Additional Files

**Supplementary_Figures.pdf**. Containing Supplementary Fig. S1 (workflow required to incorporate new tests into NApy), Supplementary Fig. S2 (runtime and memory evaluation of NApy’s C++ and Numba implementation on the NHR@FAU server), and Supplementary Fig. S3 (benchmark analysis of the impact of MAR and MNAR values per feature on the runtime of statistical tests in NApy).

**Supplementary_Listings.pdf**. Containing Supplementary Listing 1 (correlation-based hierarchical clustering using NApy and scikit-learn) and Supplementary Listing 2 (computation of principal components on correlation structures using NApy and PyTorch).

**Supplementary_Tables.xlsx**. Containing Supplementary Table S1 (runtime of NApy across features, samples, and thread counts) and Supplementary Table S2 (comparison of allocated memory usage between NApy and state-of-the-art competitors on a fixed dataset size of 1,000 features and samples with 10% missingness per feature).

**Supplementary Fig. S1**. Workflow required to incorporate new tests into NApy. To integrate a new test into NApy, researchers should fork the repository, implement the procedure either in the Python/Numba or C++ part of the code depending on compatibility, add the corresponding wrapper function in module/napy.py, and provide correctness tests in the benchmarking repository. More detailed step-by-step guidelines are available in the README of the GitHub repository.

**Supplementary Fig. S2**. Runtime and peak resident memory evaluation of NApy’s C++ and Numba implementation on the NHR@FAU server. Statistical tests were performed using varying numbers of threads on datasets comprising 1,000 samples and features with 10% of missing values per feature. (A) Fold changes between both NApy implementations were computed on the average runtime of 3 independent runs per statistical test and thread count. Cell colors represent the log_10_-transformed fold changes between NApy’s C++ and Numba implementations, while the cell labels display the corresponding nonlogarithmic fold change values as fractions. (B) For each statistical test and implementation, peak resident memory consumption (in MB) was averaged over 3 runs, with standard deviations represented as error bars.

**Supplementary Fig. S3**. Benchmark analysis of the impact of MAR and MNAR values per feature on the runtime of statistical tests in NApy. Simulated datasets with fixed sizes of 1,000 features and 1,000 samples were analyzed to evaluate the effect of varying levels of missing values per feature on runtime. All computations were performed using a single thread, and runtime measurements were averaged over 3 runs, with standard deviations represented as error bars.

**Supplementary Table S1**. Runtime of NApy across features, samples, and thread counts. The runtime (in seconds) was measured using 1, 4, 16, and 64 threads on simulated datasets varying in sample size and number of features. All datasets included 10% of missing values per feature, and the runtimes were averaged over 3 independent simulation runs. These data correspond the non-log_10_-transformed runtime values of NApy shown in Fig. 4.

**Supplementary Table S2**. Comparison of allocated memory usage between NApy and state-of-the-art competitors on a fixed dataset size of 1,000 features and samples with 10% missingness per feature. Memory consumption (in GB) for all statistical tests and libraries was averaged over 3 runs with standard deviations shown in brackets.

## Abbreviations

ANOVA: analysis of variance; CDF: cumulative distribution function; CHRIS: Cooperative Health Research in South Tyrol; MAR: missing at random; MCAR: missing completely at random; MNAR: missing not at random; SHIP: Study of Health in Pomerania.
