# Supplementary material for: NApy: efficient statistics in Python for large-scale heterogeneous data with enhanced support for missing data
Source: Gigascience. 2025 Nov 6;14:giaf140. doi: 10.1093/gigascience/giaf140 (PMC12741953; doi:10.1093/gigascience/giaf140)
Supplement: giaf140_Supplemental_Files [file giaf140_supplemental_files.zip › Supplementary_Figures.pdf]

Supplementary Figures

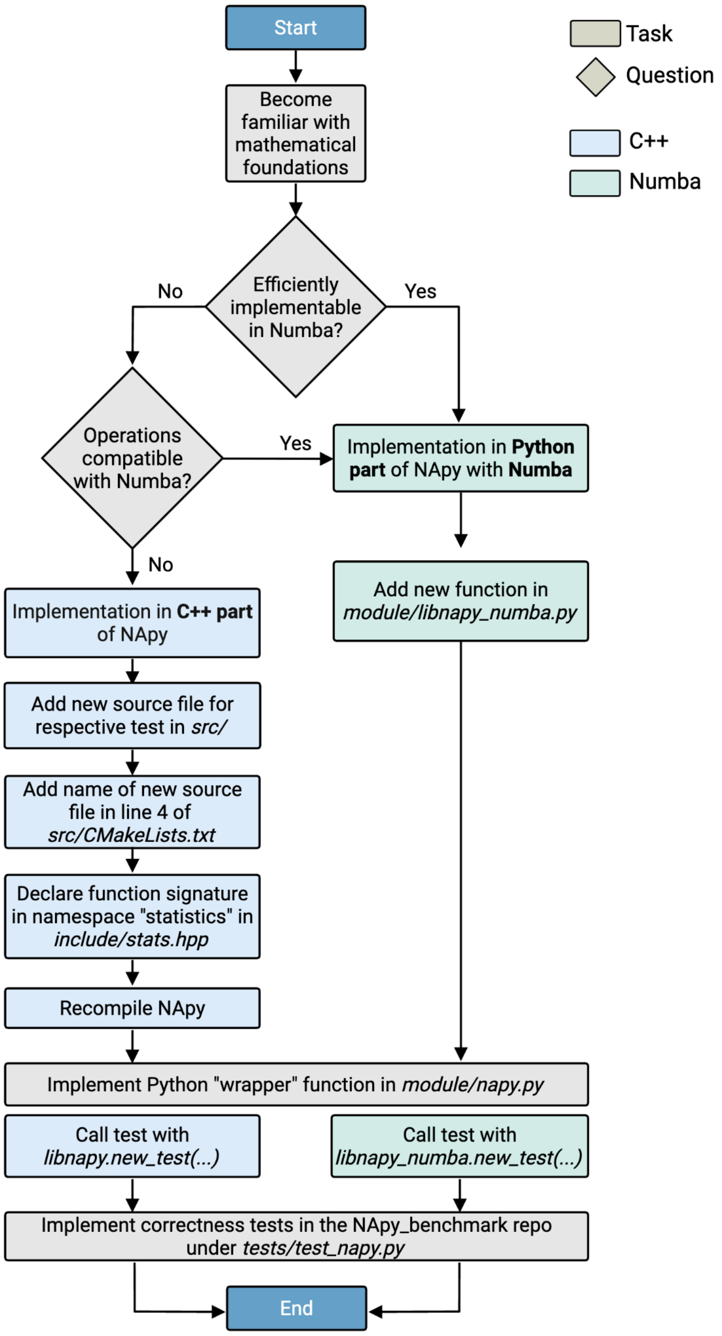

Supplementary Figure 1: Workflow required to incorporate new tests into NApy. To integrate a new test into NApy, researchers should fork the repository, implement the procedure either in the Python/Numba or C++ part of the code depending on compatibility, add the corresponding wrapper function in module/napy.py, and provide correctness tests in the benchmarking repository. More detailed step-by-step guidelines are available in the README of the GitHub repository.

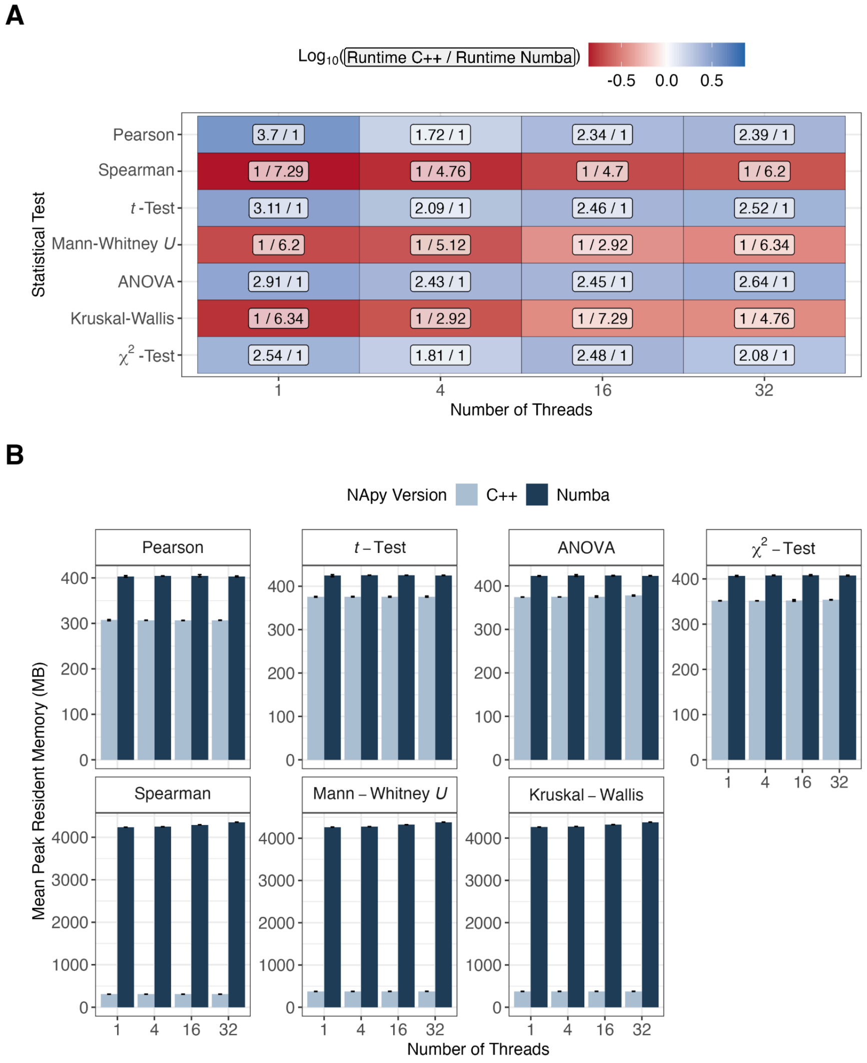

Supplementary Figure 2: Runtime and peak resident memory evaluation of NApy's C++ and Numba implementation on the NHR@FAU server. Statistical tests were performed using varying numbers of threads on datasets comprising 1000 samples and features with 10% of missing values per feature. (A) Fold changes between both NApy implementations were computed on the average runtime of three independent runs per statistical test and thread count. Cell colors represent the log10-transformed fold changes between NApy's C++ and Numba implementations, while the cell labels display the corresponding non-logarithmic fold change values as fractions. (B) For each statistical test and implementation, peak resident memory consumption (in MB) was averaged over three runs, with standard deviations represented as error bars.

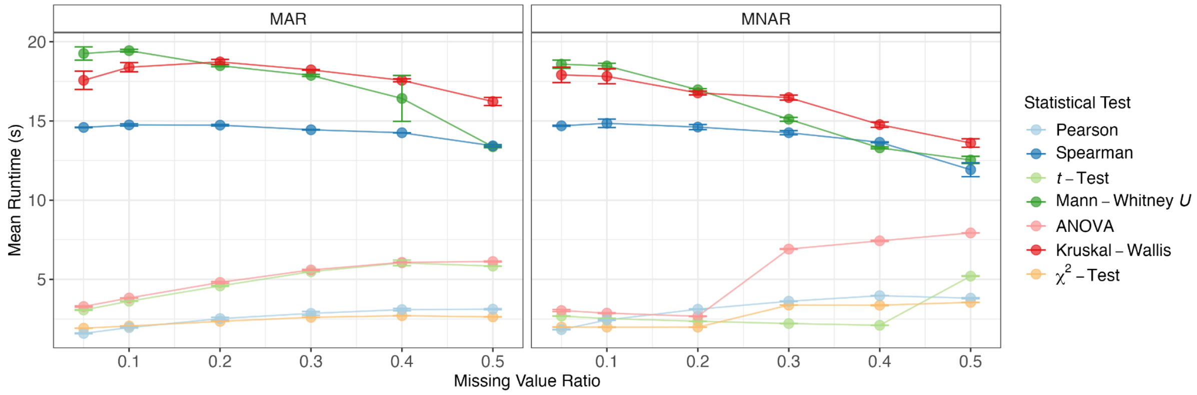

Supplementary Figure 3: Benchmark analysis of the impact of MAR and MNAR values per feature on the runtime of statistical tests in NApy. Simulated datasets with fixed sizes of 1000 features and 1000 samples were analyzed to evaluate the effect of varying levels of missing values per feature on runtime. All computations were performed using a single thread, and runtime measurements were averaged over three runs, with standard deviations represented as error bars.
