# Supplementary material for: NApy: efficient statistics in Python for large-scale heterogeneous data with enhanced support for missing data
Source: Gigascience. 2025 Nov 6;14:giaf140. doi: 10.1093/gigascience/giaf140 (PMC12741953; doi:10.1093/gigascience/giaf140)
Supplement: giaf140_Supplemental_Files [file giaf140_supplemental_files.zip › Supplementary_Listings.pdf]

Supplementary Listings

```python
import pandas as pd
import napy
from sklearn.cluster import AgglomerativeClustering

# Read numerical data.
df = pd.read_csv('example_numerical.csv', index_col=0)

# Compute NA-aware Pearson Correlation with NApy.
pearson_results = napy.pearsonr(data=df,nan_value=-99.0,threads=1)
correlation_matrix = pearson_results['r2'].to_numpy()

# Turn correlations into distances and run hierarchical clustering.
distance_matrix = 1 - correlation_matrix
clustering = AgglomerativeClustering(
    n_clusters=3,
    metric='precomputed',
    linkage='average'
)
labels = clustering.fit_predict(distance_matrix)
print(labels)
```

Supplementary Listing 1: Correlation-based hierarchical clustering using NApy and scikit-learn.

```python
import pandas as pd
import napy
import torch

# Read numerical data.
df = pd.read_csv('example_numerical.csv', index_col=0)
data_tensor = torch.tensor(df.to_numpy())

# Compute NA-aware Pearson Correlation with NApy.
spearman_results = napy.spearmanr(data=data_tensor,
                                  axis=1,
                                  nan_value=-99.0,
                                  threads=1)
correlation_tensor = spearman_results['rho']

# Eigen-decomposition of correlation matrix.
eigenvalues, eigenvectors = torch.linalg.eig(correlation_tensor)
eigenvalues = eigenvalues.real
eigenvectors = eigenvectors.real

# Sort eigenvalues in descending order.
sorted_indices = torch.argsort(eigenvalues, descending=True)
eigenvalues_sorted = eigenvalues[sorted_indices]
eigenvectors_sorted = eigenvectors[:, sorted_indices]

# Select most explanatory components.
k = 5
top_eigenvalues = eigenvalues_sorted[:k]
top_eigenvectors = eigenvectors_sorted[:, :k]

print("Top k eigenvalues:", top_eigenvalues)
print("Top k principal components:")
print(top_eigenvectors)
```

Supplementary Listing 2: Computation of principal components on correlation structures using NApy and PyTorch.
