# Supplementary material for: NApy: efficient statistics in Python for large-scale heterogeneous data with enhanced support for missing data
Source: Gigascience. 2025 Nov 6;14:giaf140. doi: 10.1093/gigascience/giaf140 (PMC12741953; doi:10.1093/gigascience/giaf140)
Supplement: giaf140_Reviewer_1_Report_Original_Submission — Feng Li -- 7/25/2025 [file giaf140_reviewer_1_report_original_submission.pdf]

Reviewer Report

Title: NApy: Efficient Statistics in Python for Large-Scale Heterogeneous Data with Enhanced Support for Missing Data

Version: Original Submission **Date:** 7/25/2025

Reviewer name: Feng Li

Reviewer Comments to Author:

The paper addresses a well-defined and important gap: most existing Python statistical packages (e.g., NumPy, SciPy, Pingouin, pandas) either lack built-in parallelization, support for pairwise missing data removal across all statistical tests, or cannot efficiently handle large heterogeneous datasets. NApy's core innovation lies in its implementation architectureâ€”a backend written in C++ with OpenMP parallelization and an alternative Numba-based backend. I have a few comments.1. I think its integration with the broader Python data science and machine learning ecosystem is currently limited. For example, frameworks like TensorFlow and PyTorch dominate in large-scale machine learning and deep learning workflows, and support for tensor-based data structures, GPU acceleration, or integration into data pipelines using tf.data or torch.utils.data would significantly enhance usability. Additionally, tighter coupling with libraries such as scikit-learn (e.g., as a statistical preprocessing or feature selection component) could make NApy more accessible to applied researchers. Providing such interfaces would increase adoption and facilitate more seamless incorporation into end-to-end machine learning workflows.2. While NApy provides an efficient solution for statistical testing on large-scale datasets within a shared-memory architecture, its scalability is inherently limited by the memory and CPU resources of a single machine. For truly large datasetsâ€”such as those exceeding tens or hundreds of gigabytes or involving millions of variablesâ€—distributed computing frameworks like Apache Spark or Dask are generally preferred. These platforms enable computation to scale across multiple nodes in a cluster, providing fault tolerance and the ability to process data that do not fit in memory. Although NApy offers substantial performance improvements within its design constraints, future extensions integrating or interoperating with distributed systems would enhance its applicability in large-scale real-world data pipelines.3. While the paper demonstrates NApy's correctness and performance under various levels of missingness, it would benefit from a more rigorous robustness check across different missing data mechanisms, specifically, Missing Completely at Random (MCAR), Missing at Random (MAR), and Missing Not at Random (MNAR). Currently, the simulations appear to introduce missingness uniformly at random (MCAR), which may not reflect real-world scenarios. Evaluating the impact of different missingness mechanisms on the validity and stability of the statistical results would strengthen the empirical evaluation and enhance the tool's credibility in practical biomedical applications.Overall, NApy is valuable practice for developers and researchers working with high-dimensional biomedical data who require scalable statistical testing with missing value support. However, the paper should be interpreted as a technical note or software paper, not a methodological advance. My above comments lie in software efficiency and usability for large datasets, not in statistical innovation.

Level of Interest

Please indicate how interesting you found the manuscript: Choose an item.

Quality of Written English

Please indicate the quality of language in the manuscript: Choose an item.

Declaration of Competing Interests

Please complete a declaration of competing interests, considering the following questions:

- Have you in the past five years received reimbursements, fees, funding, or salary from an organisation that may in any way gain or lose financially from the publication of this manuscript, either now or in the future?
- Do you hold any stocks or shares in an organisation that may in any way gain or lose financially from the publication of this manuscript, either now or in the future?
- Do you hold or are you currently applying for any patents relating to the content of the manuscript?
- Have you received reimbursements, fees, funding, or salary from an organization that holds or has applied for patents relating to the content of the manuscript?
- Do you have any other financial competing interests?
- Do you have any non-financial competing interests in relation to this paper?

If you can answer no to all of the above, write 'I declare that I have no competing interests' below. If your reply is yes to any, please give details below.

I declare that I have no competing interests

I agree to the open peer review policy of the journal. I understand that my name will be included on my report to the authors and, if the manuscript is accepted for publication, my named report including any attachments I upload will be posted on the website along with the authors' responses. I agree for my report to be made available under an Open Access Creative Commons CC-BY license (http://creativecommons.org/licenses/by/4.0/). I understand that any comments which I do not wish to be included in my named report can be included as confidential comments to the editors, which will not be published.

Choose an item.

To further support our reviewers, we have joined with Publons, where you can gain additional credit to further highlight your hard work (see: https://publons.com/journal/530/gigascience). On publication of this paper, your review will be automatically added to Publons, you can then choose whether or not to claim your Publons credit. I understand this statement.

Yes Choose an item.
