# Supplementary material for: NApy: efficient statistics in Python for large-scale heterogeneous data with enhanced support for missing data
Source: Gigascience. 2025 Nov 6;14:giaf140. doi: 10.1093/gigascience/giaf140 (PMC12741953; doi:10.1093/gigascience/giaf140)
Supplement: giaf140_Reviewer_2_Report_Original_Submission — Zane Fink, PhD -- 8/8/2025 [file giaf140_reviewer_2_report_original_submission.pdf]

Reviewer Report

Title: NApy: Efficient Statistics in Python for Large-Scale Heterogeneous Data with Enhanced Support for Missing Data

Version: Original Submission **Date:** 8/8/2025

Reviewer name: Zane Fink, PhD

Reviewer Comments to Author:

This paper presents NApy, a Python library for large-scale pairwise statistical testing in the presence of missing values. NApy is especially suitable when the datahave many missing data points. NApy's bespoke optimizations for this setting yield order-of-magnitude speedup over naive parallelization of off-the-shelf Python libraries. NApy enables real-time exploratory data analysis and could be a valuable tool during hypothesis generation.To accept the paper, I would like to see the following changes.1. A weakness is the small set (7) of implemented statistical tests. While these tests are important, and the performance results for these tests are impressive, I would like to see discussion on the required effort to implement additional tests.2. Create a stronger justification for maintaining two separate implementations (C++ with OpenMP, and Numba) for each statistical test.Doubling the implementation effort for new tests is a problem, when the advantage is unclear.Is the Numba implementation simpler? Can certain distributions of missing values cause load imbalance between threads, which OpenMP dynamic scheduling can handle?The authors observe that the Numba implementation is fasterfor parametric tests, while C++ is faster for non-parametric tests.The given explanation is conjectural; please support with profiling or microbenchmarks.Moreover, the evaluation is too limited to determine whether this is a true result or simply an artifact of the particular software and hardware used in the evaluation.3. Table 3 compares memory use of NApy to other Python libraries. NApy's parallelization scheme has little memory overhead, but the memory overhead for other schemes is not shown.The authors state: "...efficient parallelization in Python is mainly based on multiprocessing which leads to the multiplication of memory under the usage of more than one process".This claim is not supported by evidence given in the paper.While processes have heavier memory impacts than threads, multiplicative memory impact would appear only with a very poor parallelization scheme. Is this how joblib distributes the work?4. Readability would be improved by changing Figures 3 and 4a to display speedup, not its logarithm.5. The linear y-axis in Figure 4e obscures some of the differences between NApy and pandas. It would be helpful if this axis were made log scale.6. The comparison between user interfaces should be expanded to include productivity. How much effort was required to support the missing data and parallelism for competitor libraries?This would strengthen the contribution.The paper would be improved by a pass made with attention to spelling and other grammatical details. For instance, "Study of Health in Pomeria" should be "Study of Health in Pomerania".

Level of Interest

Please indicate how interesting you found the manuscript: Choose an item.

Quality of Written English

Please indicate the quality of language in the manuscript: Choose an item.

Declaration of Competing Interests

Please complete a declaration of competing interests, considering the following questions:

- Have you in the past five years received reimbursements, fees, funding, or salary from an organisation that may in any way gain or lose financially from the publication of this manuscript, either now or in the future?
- Do you hold any stocks or shares in an organisation that may in any way gain or lose financially from the publication of this manuscript, either now or in the future?
- Do you hold or are you currently applying for any patents relating to the content of the manuscript?
- Have you received reimbursements, fees, funding, or salary from an organization that holds or has applied for patents relating to the content of the manuscript?
- Do you have any other financial competing interests?
- Do you have any non-financial competing interests in relation to this paper?

If you can answer no to all of the above, write 'I declare that I have no competing interests' below. If your reply is yes to any, please give details below.

I declare that I have no competing interests.

I agree to the open peer review policy of the journal. I understand that my name will be included on my report to the authors and, if the manuscript is accepted for publication, my named report including any attachments I upload will be posted on the website along with the authors' responses. I agree for my report to be made available under an Open Access Creative Commons CC-BY license (http://creativecommons.org/licenses/by/4.0/). I understand that any comments which I do not wish to be included in my named report can be included as confidential comments to the editors, which will not be published.

Choose an item.

To further support our reviewers, we have joined with Publons, where you can gain additional credit to further highlight your hard work (see: https://publons.com/journal/530/gigascience). On publication of this paper, your review will be automatically added to Publons, you can then choose whether or not to claim your Publons credit. I understand this statement.

Yes Choose an item.
